# Attitudes toward COVID-19 vaccination and willingness to pay: comparison of people with and without mental disorders in China

**DOI:** 10.1192/bjo.2021.979

**Published:** 2021-08-11

**Authors:** Fengyi Hao, Bokun Wang, Wanqiu Tan, Syeda Fabeha Husain, Roger S. McIntyre, Xiangdong Tang, Ling Zhang, Xiaofan Han, Li Jiang, Nicholas W. S. Chew, Benjamin Yong-Qiang Tan, Bach Tran, Zhisong Zhang, Gia Linh Vu, Giang Thu Vu, Roger Ho, Cyrus S. Ho, Vijay K. Sharma

**Affiliations:** Sleep Medicine Center, Department of Respiratory and Critical Care Medicine, West China Hospital, Sichuan University, China; Mental Health Center, West China Hospital, Sichuan University, China; Translational Neuroscience Center, West China Hospital, Sichuan University, China; State Key Laboratory of Biotherapy, West China Hospital, Sichuan University, China; and Department of Psychiatry, The First People's Hospital of Chongqing Liang Jiang New Area, China; Modern Service Industry Bureau, Chongqing Liangjiang New Area Administration Committee, China; Department of Psychiatry, National University of Singapore (Chongqing) Research Institute, China; Department of Psychological Medicine, Yong Loo Lin School of Medicine, National University of Singapore, Singapore; Mood Disorders Psychopharmacology Unit, University Health Network, Ontario, Canada; Sleep Medicine Center, Department of Respiratory and Critical Care Medicine, West China Hospital, Sichuan University, China; Mental Health Center, West China Hospital, Sichuan University, China; Translational Neuroscience Center, West China Hospital, Sichuan University, China; and State Key Laboratory of Biotherapy, West China Hospital, Sichuan University, China; Department of Psychiatry, The First People's Hospital of Chongqing Liang Jiang New Area, China; Department of Psychiatry, The First People's Hospital of Chongqing Liang Jiang New Area, China; Department of Psychiatry, The First People's Hospital of Chongqing Liang Jiang New Area, China; Department of Medicine, National University Health System, Singapore; Department of Medicine, National University Health System, Singapore; Institute for Preventive Medicine and Public Health, Hanoi Medical University, Vietnam; and Bloomberg School of Public Health, Johns Hopkins University, Maryland, USA; Faculty of Education, Huaibei Normal University, China; Institute for Global Health Innovations and Faculty of Medicine, Duy Tan University, Vietnam; Center of Excellence in Behavioral Medicine, Nguyen Tat Thanh University, Vietnam; Department of Psychological Medicine, Yong Loo Lin School of Medicine, National University of Singapore, Singapore; and Institute for Health Innovation and Technology (iHealthtech), National University of Singapore, Singapore; Department of Psychological Medicine, Yong Loo Lin School of Medicine, National University of Singapore, Singapore; Division of Neurology, Department of Medicine, National University Health System, Singapore; and Department of Medicine, Yong Loo Lin School of Medicine, National University of Singapore, Singapore

**Keywords:** Anxiety, COVID-19, depression, insomnia, vaccine

## Abstract

**Background:**

Acceptance and willingness to pay for the COVID-19 vaccine are unknown.

**Aims:**

We compared attitudes toward COVID-19 vaccination in people suffering from depression or anxiety disorder and people without mental disorders, and their willingness to pay for it.

**Method:**

Adults with depression or anxiety disorder (*n* = 79) and healthy controls (*n* = 134) living in Chongqing, China, completed a cross-sectional study between 13 and 26 January 2021. We used a validated survey to assess eight aspects related to attitudes toward the COVID-19 vaccines. Psychiatric symptoms were assessed by the 21-item Depression, Anxiety and Stress Scale.

**Results:**

Seventy-six people with depression or anxiety disorder (96.2%) and 134 healthy controls (100%) reported willingness to receive the COVID-19 vaccine. A significantly higher proportion of people with depression or anxiety disorder (64.5%) were more willing to pay for the COVID-19 vaccine than healthy controls (38.1%) (*P* ≤ 0.001). After multivariate adjustment, severity of depression and anxiety was significantly associated with willingness to pay for COVID-19 vaccination among psychiatric patients (*P* = 0.048). Non-healthcare workers (*P* = 0.039), health insurance (*P* = 0.003), living with children (*P* = 0.006) and internalised stigma (*P* = 0.002) were significant factors associated with willingness to pay for COVID-19 vaccine in healthy controls.

**Conclusions:**

To conclude, psychiatric patients in Chongqing, China, showed high acceptance and willingness to pay for the COVID-19 vaccine. Factors associated with willingness to pay for the COVID-19 vaccine differed between psychiatric patients and healthy controls.

As of 31 March 2021, 130 million confirmed cases of COVID-19 infection and 2.8 million deaths have been reported by the World Health Organization.^1^ Although some people infected with COVID-19 remain asymptomatic, most patients present with fever and respiratory (sore throat, cough, nasal congestion, anosmia), gastrointestinal (nausea, vomiting) or neurological symptoms (headache), as well as myalgia and malaise.^2^ Reported long-term effects of COVID-19 infection include attention deficit, dyspnoea, fatigue, hair loss and headache.^3^ The fatality rate for COVID-19 is 1.4%.^4^

## The COVID-19 vaccine

Vaccination offers a crucial opportunity for reducing COVID-19 transmission and is a critical initiative to resolving the COVID-19 pandemic.^5^ Despite various prevailing concerns, the benefits of vaccination against COVID-19 are believed to outweigh the risks of vaccine-related side-effects, including pain and swelling at the injection site, malaise, headache, dizziness and fever.^6,7^ As of 20 March 2021, a total of 75 million COVID-19 vaccine doses have been administered.^1^ Nevertheless, several European countries, including Germany, France and Italy, temporarily paused the administration of several brands of the COVID-19 vaccines because of reports of thrombosis in some vaccine recipients.^8^ Extraordinary media attention, political discussion and public discourse may promote vaccine hesitancy among general public,^9^ and create a major public health problem during the current pandemic, leading to a huge setback to the success of the mass immunisation programme.^10^ Therefore, perception of vaccine safety is an important research topic for the COVID-19 pandemic.^11^

## The impact of the COVID-19 pandemic on mental health

The COVID-19 pandemic has led to unprecedented hazards to mental health globally.^12^ Research has confirmed the severe negative psychological impact of strict lockdown measures on people with depression or anxiety disorder.^13,14^ People with mental disorders reported higher levels of anxiety or depression compared with people without such conditions. One reason for worsening of mental disorders is the lack of access to mental health services during the pandemic.^13^ Heightened levels of anxiety, depression and insomnia could affect perception toward COVID-19 vaccination, and excessive worries could result in vaccine hesitancy. Several studies have assessed the attitude of people with mental disorders toward vaccines for various conditions. Cotugno et al reported that the overall acceptance rate of influenza vaccine in people hospitalised for psychiatric illnesses was 26%.^15^ Carney et al reported that the acceptance rates for hepatitis B, pneumococcal and influenza vaccines were 11, 30 and 45%, respectively.^16^ On the contrary, higher levels of education and perceived risk of contracting COVID-19 were associated with increased willingness to receive the COVID-19 vaccine by people suffering from multiple sclerosis.^11^ These differences in willingness to receive the COVID-19 vaccine have been studied scarcely. One study among people with substance use disorder found the lack of trust and readiness as potential barriers for COVID-19 vaccination.^17^ Other studies focused on confidence and knowledge in COVID-19 vaccines among healthcare workers.^18^ To the best of our knowledge, no study has assessed the willingness to pay for COVID-19 vaccination among people with mental disorders, including depression or anxiety disorder. In China, there are about 130 million people who suffer from mild to severe mental disorders, and 80% of them experience discrimination, thus providing a suitable population for evaluating such associations and differences.^19^

## The aims of this study

The central government of China ensures that the COVID-19 vaccines are affordable for all Chinese residents. Several places rolled out free vaccinations to key and high-risk groups, including Chongqing, Guangdong, Zhejiang and Shandong provinces. In other parts of China, people paid $35 for each shot in Wuhan^20^ and $60 for each shot in Yiwu.^21^ We used a sample of adults who suffer from depression or anxiety disorder with matched healthy controls just before the launch of the COVID-19 vaccination programme in Chongqing, a major city in China with a population of more than 30 million people. This study compared attitudes toward the pandemic and vaccines, concerns about the vaccine, perceived risk of contracting COVID-19, internalised stigma of COVID-19 infection, trust in health authorities and willingness to pay for the COVID-19 vaccine among people with depression or anxiety disorder and healthy controls. The null hypothesis was that there would be no difference in the aforementioned parameters between the two groups.

## Method

### Study design and participants

We recruited consecutive patients for the first visit or follow-up appointment in all psychiatric clinics of five hospitals in Chongqing, China, from 13 to 26 January 2021, shortly before the launch of the COVID-19 vaccination programme in the region. Four hospitals are general hospitals and one hospital is a psychiatric hospital. All hospitals are public hospitals run by the city and provincial health authorities. A hard copy of questionnaire was administered after obtaining a written consent from participants. A group of age-, gender- and education-matched healthy controls were recruited through posters on the noticeboards of the hospitals and word of mouth in Chongqing between 15 and 27 January 2021. All procedures involved in this study complied with the ethical standards of the relevant national and institutional committees on human experimentation and the Declaration of Helsinki. The study was approved by Ethics Review Committee of The First People's Hospital of Chongqing Liang Jiang New Area (approval number 2021-01-006).

### Inclusion and exclusion criteria

All people with depression or anxiety disorder were aged ≥18 years with psychiatrist-diagnosed major depressive disorder (single episode or recurrent episodes) or anxiety disorders including generalised anxiety disorder and panic disorder, based on the ICD-10 criteria (codes F32, F33 and F41, respectively). Healthy controls were aged ≥18 years with no history of psychiatric illnesses.

### Measures

This study used the National University of Singapore COVID-19 vaccine questionnaire that was developed and validated in the Asia-Pacific regions, including China, India, Indonesia, Bhutan, Singapore and Vietnam.^22^ Participants indicated their responses in eight aspects. The first aspect was participant's willingness to receive the vaccine. This was assessed by a single item: ‘I would be willing to receive the COVID-19 vaccine if it was safe, available and recommended’. Willingness to receive the COVID-19 vaccine was dichotomised (1, willing; 0, not willing). The second aspect was perception of the COVID-19 pandemic and vaccine. A total of three items assessed the perception of pandemic and the vaccine. The third aspect was participant's concerns of the vaccine. This was subdivided into three components: (a) physical harm index (five items) assessing the participant's perception of the anticipated harm by the vaccine, (b) financial concerns related to the vaccine (one item) and (c) other concerns (four items). The fourth aspect was the COVID-19 risk profile. This included nine items assessing the direct impact of COVID-19 infection on their personal, family and social life.

The fifth aspect was internalised stigma of COVID-19 infection. The internalised stigma of COVID-19 infection questionnaire was adapted from the Perceived External Stigma of the Ebola-related Stigma Questionnaire,^23^ and was assessed by three items: ‘I would feel stigmatised if I contracted COVID-19’, ‘I am worried that others may refuse to have contact with me If I were to receive the vaccine’ and ‘I am worried that others may think that I have COVID-19 if I were to receive the vaccine’. This assessed participants’ internalised stigma of receiving the COVID-19 vaccine. The sixth aspect was a pro-socialness scale, evaluating participants’ pro-socialness stance on the COVID-19 vaccine, including social responsibility of vaccination and agreement to receive other vaccines (e.g. influenza vaccines).

The seventh aspect was public trust of health authorities. This evaluated the extent of the public's trust in the healthcare and government sector during the pandemic. Five items encompassing various components of the public's trust (including participants’ overall perception of trust in health authorities and perception of authorities’ competency, fairness, honesty and confidentiality). A Likert scale was used to assess participants’ responses (1, strongly agree; 2, agree; 3, neutral; 4, disagree; 5, strongly disagree). Consistent with previous literature using the vaccine willingness scale, items with the same index were collapsed into a composite score for analysis in the logistic model.^24^ The eighth aspect was related to participants’ willingness to pay for the vaccination.

Mental health status was assessed with the 21-item Depression, Anxiety and Stress Scale (DASS-21). The DASS-21 was demonstrated to be a reliable and valid measure in assessing mental health in the Chinese population and people with depression or anxiety disorder during the COVID-19 pandemic.^13,25^ Based on previous COVID-19 research, the Cronbach's alpha for the subscales of the Chinese version of DASS-21 was >0.8, indicating high internal consistency in assessing stress, depression and anxiety in the Chinese population.^26^

### Statistical analysis

The effect of psychiatric diagnosis on continuous and categorical variables was determined with the Student's *t*-test and Pearson's *χ*²-test, respectively. Variables included demographic characteristics, socioeconomic status, occupation, medical history and subsections of the questionnaire. Associations between questionnaire measures and willingness to pay for the vaccine were determined with univariate binary logistic regression analysis. Questionnaire measures that were associated with willingness to pay for the vaccine on univariate analysis were included in a subsequent multivariable binary logistic regression model. The dependent variable was willingness to pay for the vaccine, and covariates were healthcare worker status, ownership of private health insurance, presence of children or dependents, presence of medical condition, perceived risk index, physical harm index, financial concern, personal risk profile, internalised stigma, pro-socialness index, public trust in health authorities and DASS-21 score. All tests were two-tailed, with a significance level of *P* ≤ 0.05. Data are expressed as mean (s.d.). Statistical analysis was performed with SPSS Statistics version 26 for Windows (IBM Corporation).

## Results

A total of 90 people with mental disorders were approached; 7 people refused and 8 did not complete the questionnaire. The response rate for people with depression or anxiety disorder was 83.33% (*n* = 75). Out of 162 healthy controls, 28 refused to participate after obtaining more information on the study. The response rate for healthy controls was 82.71% (*n* = 134). Table 1 summarises the characteristics of participants. Of the 213 participants included in the final analyses, there were 79 people with mental disorders and 134 healthy controls. Out of 79 people with mental disorders, 56 people suffered from F33 major depressive disorder and 23 people suffered from F41 anxiety disorder.

**Table 1:** Characteristics of people with depression or anxiety disorder and healthy controls (*N* = 213)

Variable	People with depression or anxiety disorder (*n* = 79)	Healthy controls (*n* = 134)	*P*-value
Age, years^a^	33.8 ± 14.3	35.7 ± 13.4	0.328
Gender^b^			0.795
Male	24 (30.4%)	43 (32.1%)	
Female	55 (69.6%)	91 (67.9%)	
Marital status^b^			0.382
Single	34 (43%)	50 (37.3%)	
Married	37 (46.8%)	75 (56%)	
Separated, divorced or widowed	8 (10.1%)	9 (6.7%)	
Employed during the COVID-19 pandemic^b^^,^^c^	40 (50.6%)	100 (75.2%)	**≤0.001**
Occupation^b^^,^^c^			**≤0.001**
Government	16 (20.5%)	14 (10.4%)	
Private or self-employed	26 (33.3%)	89 (66.4%)	
Retired	6 (7.7%)	12 (9%)	
Student	22 (28.2%)	13 (9.7%)	
Not employed	8 (10.3%)	6 (4.5%)	
Healthcare worker as occupation^b^	4 (5.1%)	61 (45.5%)	**≤0.001**
Smoking status^b^			0.828
Current smoker	13 (16.5%)	18 (13.4%)	
Ex-smoker	6 (7.6%)	10 (7.5%)	
Non-smoker	60 (75.9%)	106 (79.1%)	
Housing^b^			0.160
One-room flat	8 (10.1%)	8 (6%)	
Two-room flat	26 (32.9%)	37 (27.6%)	
Three-room flat	31 (39.2%)	75 (56%)	
Four-room flat	8 (10.1%)	9 (6.7%)	
Five-room flat, condominium or landed (property or houses)	6 (7.6%)	5 (3.7%)	
Annual household income^b^			**≤0.001**
<$25 000	37 (46.8%)	92 (68.7%)	
$25 000–$34 999	19 (24.1%)	27 (20.1%)	
≥$35 000	23 (29.1%)	15 (11.2%)	
Education level^b^			0.592
No education or less than high school	3 (3.8%)	9 (6.7%)	
High school	20 (25.3%)	37 (27.6%)	
Undergraduate or postgraduate	56 (70.9%)	88 (65.7%)	
Chronic medical condition^b^	37 (46.8%)	21 (15.7%)	**≤0.001**
On long-term medication^b^	43 (54.4%)	13 (9.7%)	**≤0.001**
Private health insurance^b^	29 (36.7%)	38 (28.4%)	0.205
Children or dependents^b^	33 (41.8%)	74 (55.2%)	0.058
Religious^b^	10 (12.7%)	13 (9.7%)	0.502
Exercise^b^	21 (26.6%)	23 (17.2%)	0.101
Psychiatric diagnosis			−
Depression	56 (42.7%)	−	
Generalised anxiety disorder	23 (17.6%)	−	
DASS-21 score^a^	14.2 ± 11.4	2.6 ± 3.7	**≤0.001**

*P* ≤ 0.05 indicated in bold. DASS-21, 21-item Depression, Anxiety and Stress Scale.

a. Student's *t*-test.

b. Pearson's *χ*²-test.

c. Complete data was not obtained (occupation: healthy controls, *n* = 134; people with depression or anxiety disorder, *n* = 78; employed during the COVID-19 pandemic: healthy controls, *n* = 133; people with depression or anxiety disorder, *n* = 79.)

There were no significant differences in age, gender, marital status, smoking status, housing ownership of private health insurance and education level between people with depression or anxiety disorder and healthy controls (*P* > 0.05). The participants were predominantly young adults (mean age of people with depression or anxiety disorder, 33.8±14.3 years; mean age of healthy controls, 35.7±13.4 years), female (people with depression or anxiety disorder, 69.6%; healthy controls, 67.9%), non-smokers (people with depression or anxiety disorder, 75.9%; healthy controls, 79.1%) and university educated (people with depression or anxiety disorder, 70.9%, healthy controls, 65.7%). There were significantly more healthy controls who were employed during the COVID-19 pandemic than people with depression or anxiety disorder (76.8 *v*. 53.8%, *P* = 0.027), but people with depression or anxiety disorder had significantly higher income than healthy controls (≥$35 000 per year, 29.1 *v*. 11.2%; *P* ≤ 0.001). There were significantly more people with depression or anxiety disorder who had chronic medical conditions (46.8 *v*. 15.7%, *P* ≤ 0.001) and were on long-term medication (54.4 *v*. 9.7%, *P* ≤ 0.001). People with depression or anxiety disorder had significantly higher DASS-21 scores (14.2 *v.* 2.6, *P* ≤ 0.001).

Supplementary Table 1 available at https://doi.org/10.1192/bjo.2021.979 compares the responses for each question in the questionnaire. The acceptance rate for the COVID-19 vaccine was 100% for healthy controls and 96.2% for people with depression or anxiety disorder. Healthy controls had a significantly higher acceptance rate (*P* = 0.023). Significantly higher proportion of people with depression or anxiety disorder were concerned about having to sign informed consent documents than healthy controls (27.8 *v*. 24.6%, *P* = 0.021). More healthy controls were worried about the cost or inability to afford the COVID-19 vaccine than people with depression or anxiety disorder (9 *v*. 1.3%, *P* = 0.049), and more healthy controls expected the COVID-19 vaccine to be free of charge (61.9 *v.* 35.4%, *P* ≤ 0.001). In contrast, more people with depression or anxiety disorder (13.9%) were willing to pay >$250 for the COVID-19 vaccine than healthy controls (5.2%, *P* ≤ 0.001), whereas more healthy controls expected the COVID-19 vaccine to be provided at no cost (61.9 *v.* 35.4%, *P* ≤ 0.001). A significantly higher proportion of people with depression or anxiety disorder strongly felt that the public healthcare system was effective (53.2 *v.* 39.6%, *P* = 0.039) than healthy controls. There were no differences between people with depression or anxiety disorder and healthy controls in other questions, including stigma after contracting of COVID-19, safety concerns over vaccines, willingness to receive other vaccines, outlook of vaccine leading to normal life and intention to travel (*P* > 0.05).

Table 2 compares the scores of eight subscales of the COVID-19 vaccine questionnaire between people with depression or anxiety disorder and healthy controls. People with depression or anxiety disorder were more likely to pay for the COVID-19 vaccine (64.6 *v.* 38.1%, *P* ≤ 0.001) and showed significantly higher levels of physical harm concerns (14.7 *v.* 13.8%, *P* = 0.027) and financial concerns regarding the COVID-19 vaccine (5.6% *v.* 4.6%, *P* ≤ 0.001). People with depression or anxiety disorder reported higher score in the personal risk profile (0.8 *v*. 0, *P* ≤ 0.001) and family risk (0.4 *v.* 0.2, *P* = 0.047), compared with healthy controls. There were no significant differences between people with depression or anxiety disorder and healthy controls in the perception of the COVID-19 vaccines and other concerns associated with risk of contracting COVID-19, overall perceived risk index, internalised stigma of potential COVID-19 infection, pro-socialness stance on the vaccine and trust in health authorities between people with depression or anxiety disorder and healthy controls (*P* > 0.05).

**Table 2:** Comparison of subscale scores between people with depression or anxiety disorder and healthy controls (*N* = 213)

Variable	People with depression or anxiety disorder (*n* = 79)	Healthy controls (*n* = 134)	*P*-value
Willing to pay for the COVID-19 vaccine^a^	51 (64.6%)	51 (38.1%)	**≤0.001**
Perception of the COVID-19 pandemic and vaccines^b^	8.8 ± 2.3	8.5 ± 2	0.303
Concerns relating to the vaccine			
Physical harm index^b^	14.7 ± 2.9	13.8 ± 2.9	**0.027**
Financial concerns^b^	5.6 ± 1.6	4.6 ± 1.7	**≤0.001**
Other concerns^b^	12.1 ± 2.2	11.9 ± 1.9	0.572
COVID-19 risk profile			
Personal^b^	0.8 ± 0.4	0 ± 0.2	**≤0.001**
Family^b^	0.4 ± 0.5	0.2 ± 0.4	0.047
Social life^b^	0 ± 0.2	0 ± 0	0.159
Internalised stigma of COVID-19 infection^b^	10.4 ± 1.8	10.3 ± 1.7	0.617
Pro-socialness scale^b^	8.7 ± 2.3	8.6 ± 2.4	0.777
Public trust in health authorities^b^	9.3 ± 3	9.9 ± 3.3	0.171
Perceived risk index^b^	8.5 ± 2.1	8.3 ± 2	0.454

*P* ≤ 0.05 indicated in bold.

a. Pearson's *χ*²-test.

b. Student's *t*-test.

Table 3 shows the univariate and multivariate logistic regression between willingness to pay for the COVID-19 vaccine and independent variables among people with depression or anxiety disorder. Personal risk profile of contracting COVID-19 (*P* = 0.047) and DASS-21 scores (*P* = 0.014) were independently associated with higher willingness to pay for the COVID-19 vaccine. After multivariate adjustment, higher DASS-21 score was significantly associated with willingness to pay for COVID-19 vaccination (*P* = 0.048). The regression model captured 13.9% variance in willingness to pay for the COVID-19 vaccine.

**Table 3:** Univariate and multivariate logistic analysis of willingness to pay for COVID-19 vaccination among people with depression or anxiety disorder (*n* = 79)

Variable	Odds ratio (95% CI)	*P*-value	Adjusted odds ratio (95% CI)	*P*-value
Non-healthcare workers	6.000 (0.593−60.660)	0.129	−	−
Coverage by private health insurance	0.734 (0.278−1.914)	0.533	−	−
Living with children or dependents	1.071 (0.421−2.725)	0.885	−	−
Presence of medical condition	0.975 (0.387−2.457)	0.957	−	−
Perceived risk index	1.022 (0.824−1.269)	0.841	−	−
Physical harm index	1.052 (0.898−1.232)	0.529	−	−
Personal risk profile	2.986 (1.014−8.796)	**0.047**	2.004 (0.620−6.475)	0.246
Internalised stigma of COVID-19 infection	1.277 (0.973−1.676)	0.078	−	−
Pro-socialness index	1.002 (0.815−1.231)	0.988	−	−
Public trust in health authorities	1.113 (0.947−1.308)	0.195	−	−
DASS-21 score	1.063 (1.012−1.117)	**0.014**	1.052 (1.000−1.107)	**0.048**

Healthcare workers, those with private health insurance, those with children or dependents, those with a medical condition and those who were willing to pay for the vaccine were coded ‘1’ and were the reference category (last). DASS-21, 21-item Depression, Anxiety and Stress Scale.

Table 4 shows the univariate and multivariate logistic regression between willingness to pay for the COVID-19 vaccine and independent variables among healthy controls. Non-healthcare worker status (*P* ≤ 0.001), private health insurance (*P* ≤ 0.001), living with children or dependents (*P* ≤ 0.001), physical harm index (*P* ≤ 0.001), personal risk profile of contracting COVID-19 (*P* ≤ 0.049)**,** internalised stigma of COVID-19 infection (*P* ≤ 0.001), pro-socialness index (*P* = 0.004) and DASS-21 scores (*P* ≤ 0.001) were independently associated with higher willingness to pay for the COVID-19 vaccine. After multivariate adjustment, non-healthcare worker status (*P* = 0.039), private health insurance (*P* = 0.003), living with children or dependents (*P* = 0.006) and internalised stigma of COVID-19 infection (*P* = 0.002) were significantly associated with willingness to pay for COVID-19 vaccination. The regression model captured 30.6% variance in willingness to pay for the COVID-19 vaccine.

**Table 4:** Univariate and multivariable binomial logistic regression of willingness to pay for COVID-19 vaccination among healthy controls (*n* = 134)

Variable	Odds ratio (95% CI)	*P*-value	Adjusted odds ratio (95% CI)	*P*-value
Non-healthcare worker status	7.828 (3.358−18.246)	**≤0.001**	3.369 (1.064−10.667)	**0.039**
Coverage by private health insurance	0.228 (0.103−0.506)	**≤0.001**	0.128 (0.034−0.486)	**0.003**
Living with children or dependents	3.802 (1.823−7.933)	**≤0.001**	5.733 (1.657−19.829)	**0.006**
Presence of medical condition	0.498 (0.195−1.274)	0.146	−	−
Perceived risk index	0.929 (0.779−1.108)	0.415	−	−
Physical harm index	1.279 (1.106−1.480)	**≤0.001**	1.105 (0.903−1.353)	0.333
Personal risk profile	8.913 (1.010−78.625)	**0.049**	1.137 (0.064−20.223)	0.930
Internalised stigma of COVID-19 infection	2.238 (1.621−3.089)	**≤0.001**	1.880 (1.267−2.788)	**0.002**
Pro-socialness index	0.797 (0.681−0.931)	**0.004**	0.863 (0.696−1.071)	0.181
Public trust in health authorities	0.911 (0.816−1.017)	0.096	−	−
DASS-21 score	1.226 (1.097−1.370)	**≤0.001**	1.114 (0.957−1297)	0.163

Healthcare workers, those with private health insurance, those with children or dependents, those with a medical condition and those who were willing to pay for the vaccine were coded ‘1’ and were the reference category (last). DASS-21, 21-item Depression, Anxiety and Stress Scale.

## Discussion

The key finding of this study is that people with and without mental disorders showed a high acceptance rate for the COVID-19 vaccine in Chongqing, China. The higher willingness to pay for the vaccine by people with depression or anxiety disorder is a novel finding. Factors associated with willingness to pay for COVID-19 vaccination differed between people with and without mental disorders. For people with depression or anxiety disorder, high DASS-21 score was associated with willingness to pay for the COVID-19 vaccine. In contrast, non-healthcare worker status, having private health insurance, living with children or dependents and internalised stigma of COVID-19 infection were associated with willingness to pay for the COVID-19 vaccine in healthy controls. Our findings provide health authorities with information regarding the attitudes of people with depression or anxiety disorder regarding COVID-19 vaccination. The high acceptance rate among people with depression or anxiety disorder and healthy controls is encouraging for the aim of achieving herd immunity.

The high proportion of willingness to receive the COVID-19 vaccine in people with depression or anxiety disorder is similar to our observation among healthcare workers across Asia.^22^ Interestingly, the willingness to receive vaccination in our study is higher than recent studies conducted in the general population in China (84.8–91.3%),^27,28^ as well as among multiple sclerosis patients in the USA (66%).^11^ Similarly, the acceptance rate for the healthy controls in this study is higher than general population in the USA (67%) and Japan (65.7%).^29,30^ Importantly, our study was conducted before the launch of the COVID-19 vaccination programme in Chongqing, China, in contrast to the prior USA study conducted in 2020, when the COVID-19 vaccine was hypothetical.^11^ By January 2021, China had made progress in developing several COVID-19 vaccines,^31^ and about 64% of Chinese had even expressed a preference for a China-made vaccine over foreign-made vaccine.^32^ By 20 March 2021, China had administered 75 million doses of COVID-19 vaccines.^33^ Since vaccine willingness might change over time as more data is accumulated about the safety and efficacy of the vaccine, further study may be required to monitor the trend worldwide.

The other explanation for the high COVID-19 vaccine acceptance rate in our study is the high confidence in the public healthcare system and recommendations from the Chinese Government. In our study, only 3.7% of healthy controls and 3.8% of people with depression or anxiety disorder expressed disagreement or strong disagreement that the COVID-19 vaccine was safe. This is in contrast to a study in France in which 25% of the adult participants refused to take the COVID-19 vaccine, citing concerns over its safety.^34^ Furthermore, the high acceptance rate in our study could be because Chinese people are more collectivistic, attached to social conformity and responsive to administrative collective orders such as wearing face masks during the current pandemic.^35^ A similar attitude was responsible for the high acceptance rate noted among Asian Americans among all racial and ethnic groups in a recent study in the USA,^29^ which might explain the better containment of the current pandemic in some Asia countries.

Previous studies found that education levels and perceived risk of COVID-19 infection were associated with willingness to receive the COVID-19 vaccine.^11,27^ Our study found that internalised stigma was significantly associated with willingness to pay for COVID-19 vaccination, after multivariate adjustment in healthy controls. Our finding has important implications for the community and supports the previous postulation that demographics, health status and source of COVID-19 information do not completely explain willingness to pay for the COVID-19 vaccine.^11^ Physician- or pharmacist-led programmes for vaccine education and immunisation counselling might further address the benefits and risk of vaccination, the potential interaction with medications, the differences between somatic complaints and vaccine side-effects, and improve compliance. Interestingly, for healthy controls, internalised stigma associated with COVID-19 infection was a motivator, not a barrier, to paying for the vaccine and thereby reducing the risk of contracting COVID-19. This is in contrast to a previous study in which the stigma associated with an infectious disease increased vaccine hesitancy and resistance.^36^ For people with depression or anxiety disorder, high DASS-21 score was the only factor associated with willingness to pay for the COVID-19 vaccine, after adjustment for other factors. Psychiatric patients might be more depressed and anxious during the pandemic, and this might explain their keenness for vaccination.

Although this study found that a third of people with depression or anxiety disorder, and two-thirds of healthy controls, preferred to receive free COVID-19 vaccines, there was a significantly higher proportion of people with depression or anxiety disorder who were willing to pay for the COVID-19 vaccine and had fewer financial concerns. There could be several explanations for this finding. First, depression and anxiety disorders are mild psychiatric conditions and have less impact on occupational function and willingness to pay for the COVID-19 vaccine. In this study, about 53.8% of people with depression or anxiety disorder and 76.8% of healthy controls were employed. Second, the proportion of healthcare workers was higher in healthy controls (45.5%) compared with people with depression or anxiety disorder (5.1%). Among healthy controls, non-healthcare worker status was a significant factor associated with willingness to pay for the COVID-19 vaccine. We performed further analysis by comparing annual incomes of people with depression or anxiety disorder, healthy controls who were healthcare workers and healthy controls who were not healthcare workers. In this study, 28% of people with depression or anxiety disorder, 15.1% of healthy controls who were not healthcare workers and 6.6% of healthy controls who were healthcare workers reported an annual income ≥$35 000, and the differences were significant (*P* = 0.005). The Chinese government has maintained its pricing power over healthcare services and salaries.^37^ As a result, China's healthcare workers are underpaid compared with other professions. Furthermore, healthcare workers might expect the vaccine to be free of charge, which could affect their willingness to pay for COVID-19 vaccine. Our finding is different when compared with a recent study that reported that median willingness to pay for the COVID-19 vaccine was $28 in China.^32^ In our study, >25% of people with depression or anxiety disorder and healthy controls were willing to pay $50 for the COVID-19 vaccine. More than 5% of people with depression or anxiety disorder and healthy controls were willing to pay $250 for the COVID-19 vaccine.

Our findings need to be interpreted in context of the economic status of Chongqing, where this study was conducted. The gross domestic product of Chongqing in 2020 was 2 500 279 billion yuan ($380.925 billion), ranking fifth among all cities in China. As a result, some people with depression or anxiety disorder and healthy controls were able to afford to pay for the COVID-19 vaccine. The Chinese healthcare system has a vibrant private sector and patients may engage both public and private health services. Many Chinese with mental disorders pay for their psychiatric treatment as part of the chronic disease management, which might explain why people with depression or anxiety disorder in our study were more willing pay for the COVID-19 vaccine compared with healthy controls, who were willing to pay if covered by health insurance. Our findings may not apply to other cities with a lower gross domestic product, and further research in other countries is required.

### Limitations

Although this study is among the first to report the willingness to pay for the COVID-19 vaccine in people with depression or anxiety disorder, some limitations need to be acknowledged. First, we studied people without psychosis, and mainly focused on people suffering from depression or anxiety disorder. These patients might have better occupational potential and higher socioeconomic status than people with psychotic illnesses (e.g. schizophrenia), although people with depression or anxiety disorder are prone to cognitive bias that may affect perception of the COVID-19 vaccines.^38^ Furthermore, patients with psychosis experience higher levels of stigma compared with those without psychosis.^19^ Our findings could not be generalised to people suffering from severe mental disorders (e.g. schizophrenia) that result in severe financial difficulty, as well as those with low educational background. Furthermore, we measured anxiety and depression symptoms in general, and we did not assess the potential effects of COVID-19-related anxiety as a specific cause of vaccination willingness.

Second, our study participants were from one city in China, and the findings may not be generalized to the rest of China and other countries. Third, the regression model captured 30.6% variance in willingness to pay for the COVID-19 vaccine in people with depression or anxiety disorder, suggesting that other factors were not assessed in this study. Nevertheless, the percentage of variance is higher than previous studies on willingness to receive the COVID-19 vaccine.^11^ Fourth, the study design was cross-sectional and could not demonstrate causal relationship between independent variables and willingness to pay for the COVID-19 vaccines. Fifth, the study was based on self-administered questionnaires, where the responses could be affected by recall and reporting bias.

To conclude, >95% of people with depression or anxiety disorder and healthy controls were willing to receive the COVID-19 vaccine in Chongqing, China. People with depression or anxiety disorder were more willing to pay for the COVID-19 vaccine than healthy controls. Severity of depression and anxiety was the most important factor associated with willingness to pay for the COVID-19 vaccine in people with depression or anxiety disorder. In contrast, non-healthcare worker status, ownership of private insurance, living with children and adolescents and internalised stigma associated with COVID-19 infection were the most important factors associated with willingness to pay for the COVID-19 vaccine in healthy controls. As people with mental disorders are keen to accept the COVID-19 vaccine, health authorities should offer vaccination as soon as possible.

## Data Availability

The data that support the findings of this study are available on request from the corresponding author, R.H.. The data are not publicly available due to their containing information that could compromise the privacy of research participants.

## Supplementary material

For supplementary material accompanying this paper visit http://dx.doi.org/10.1192/bjo.2021.979. click here to view supplementary material
