# Supplementary material for: Attitudes toward COVID-19 vaccination and willingness to pay: comparison of people with and without mental disorders in China
Source: BJPsych Open. 2021 Aug 11;7(5):e146. doi: 10.1192/bjo.2021.979 (PMC8365102; doi:10.1192/bjo.2021.979)
Supplement: Supplementary file 1 [file S2056472421009790sup001.docx]

**Supplementary Table 1.** Responses for the individual items of the COVID-19 vaccine questionnaires **(**Perceptions (B1-B19), hesitancy factors (D1-12) and other factors (E1-11)). (N = 213)

| Question | Response | Healthy controls | Psychiatric Patients | *p*-value |
| --- | --- | --- | --- | --- |
| 1. The current COVID-19 pandemic is severe | Strongly agree | 26 (19.4%) | 20 (25.3%) | 0.432 |
|  | Agree | 52 (38.8%) | 27 (34.2%) |  |
|  | Neutral | 48 (35.8%) | 24 (30.4%) |  |
|  | Disagree | 8 (6%) | 7 (8.9%) |  |
|  | Strongly disagree | 0 | 1 (1.3%) |  |
| 1. I am at risk of contracting COVID-19 | Strongly agree | 7 (5.2%) | 6 (7.6%) | 0.810 |
|  | Agree | 25 (18.7%) | 18 (22.8%) |  |
|  | Neutral | 52 (38.8%) | 25 (31.6%) |  |
|  | Disagree | 42 (31.3%) | 25 (31.6%) |  |
|  | Strongly disagree | 8 (6%) | 5 (6.3%) |  |
| 1. I would feel stigmatised if I contracted COVID-19 | Strongly agree | 12 (9%) | 8 (10.1%) | 0.962 |
|  | Agree | 32 (23.9%) | 22 (27.8%) |  |
|  | Neutral | 47 (35.1%) | 26 (32.9%) |  |
|  | Disagree | 35 (26.1%) | 19 (24.1%) |  |
|  | Strongly disagree | 8 (6%) | 4 (5.1%) |  |
| 1. It is my social responsibility to ensure that I am adequately protected/vaccinated against COVID-19 | Strongly agree | 71 (53%) | 44 (55.7%) | 0.901 |
|  | Agree | 41 (30.6%) | 25 (31.6%) |  |
|  | Neutral | 17 (12.7%) | 8 (10.1%) |  |
|  | Disagree | 5 (3.7%) | 2 (2.5%) |  |
|  | Strongly disagree | 0 | 0 |  |
| 1. I have trust in my country’s public healthcare system/government | Strongly agree | 55 (41%) | 38 (48.1%) | 0.185 |
|  | Agree | 58 (43.3%) | 34 (43%) |  |
|  | Neutral | 21 (15.7%) | 6 (7.6%) |  |
|  | Disagree | 0 | 1 (1.3%) |  |
|  | Strongly disagree | 0 | 0 |  |
| 1. My country’s public healthcare system/government has responded effectively to the COVID-19 pandemic | Strongly agree | 53 (39.6%) | 42 (53.2%) | **0.039** |
|  | Agree | 58 (43.3%) | 31 (39.2%) |  |
|  | Neutral | 23 (17.2%) | 5 (6.3%) |  |
|  | Disagree | 0 | 1 (1.3%) |  |
|  | Strongly disagree | 0 | 0 |  |
| 1. I am confident that my country’s public healthcare system/government will respond fairly to my health needs, regardless of my race, ethnicity, income or other personal characteristics | Strongly agree | 37 (27.6%) | 24 (30.4%) | 0.787 |
|  | Agree | 49 (36.6%) | 27 (34.2%) |  |
|  | Neutral | 36 (26.9%) | 24 (30.4%) |  |
|  | Disagree | 11 (8.2%) | 4 (5.1%) |  |
|  | Strongly disagree | 1 (0.7%) | 0 |  |
| 1. My country’s public healthcare system/government has provided honest information/been transparent about the COVID-19 pandemic to the public | Strongly agree | 31 (23.1%) | 22 (27.8%) | 0.669 |
|  | Agree | 63 (47%) | 39 (49.4%) |  |
|  | Neutral | 34 (25.4%) | 16 (20.3%) |  |
|  | Disagree | 6 (4.5%) | 2 (2.5%) |  |
|  | Strongly disagree | 0 | 0 |  |
| 1. I am confident that the personal data (such as my race, income and citizenship) collected by my country’s government will not be used against me | Strongly agree | 29 (21.6%) | 22 (27.8%) | 0.206 |
|  | Agree | 67 (50%) | 38 (48.1%) |  |
|  | Neutral | 35 (26.1%) | 14 (17.7%) |  |
|  | Disagree | 3 (2.2%) | 5 (6.3%) |  |
|  | Strongly disagree | 0 | 0 |  |
| 1. I am worried about the cost of/unable to afford the COVID-19 vaccine | Strongly agree | 12 (9%) | 1 (1.3%) | **0.049** |
|  | Agree | 33 (24.6%) | 16 (20.3%) |  |
|  | Neutral | 40 (29.9%) | 19 (24.1%) |  |
|  | Disagree | 38 (28.4%) | 33 (41.8%) |  |
|  | Strongly disagree | 11 (8.2%) | 10 (12.7%) |  |
| 1. The maximum amount of money (USD) I am willing to spend on the COVID-19 vaccine | Free of charge | 83 (61.9%) | 28 (35.4%) | **≤0.001** |
|  | 50 | 36 (26.9%) | 20 (25.3%) |  |
|  | 100 | 8 (6%) | 20 (25.3%) |  |
|  | ≥250 | 7 (5.2%) | 11 (13.9%) |  |
| 1. I belief that vaccines in general are safe and effective | Strongly agree | 19 (14.2%) | 13 (16.5%) | 0.245 |
|  | Agree | 48 (35.8%) | 37 (46.8%) |  |
|  | Neutral | 62 (46.3%) | 26 (32.9%) |  |
|  | Disagree | 3 (2.2%) | 3 (3.8%) |  |
|  | Strongly disagree | 2 (1.5%) | 0 |  |
| 1. I am willing to receive other vaccines, eg the influenza vaccine | Strongly agree | 25 (18.7%) | 12 (15.2%) | 0.099 |
|  | Agree | 56 (41.8%) | 43 (54.4%) |  |
|  | Neutral | 45 (33.6%) | 16 (20.3%) |  |
|  | Disagree | 8 (6%) | 8 (10.1%) |  |
|  | Strongly disagree | 0 | 0 |  |
| 1. I am intending to/have already been vaccinated against the flu | Strongly agree | 14 (10.4%) | 8 (10.1%) | 0.082 |
|  | Agree | 32 (23.9%) | 16 (20.3%) |  |
|  | Neutral | 66 (49.3%) | 29 (36.7%) |  |
|  | Disagree | 21 (15.7%) | 24 (30.4%) |  |
|  | Strongly disagree | 1 (0.7%) | 2 (2.5%) |  |
| 1. How much do you feel that the COVID-19 pandemic has affected your daily life? | Strongly agree | 17 (12.7%) | 5 (6.3%) | 0.114 |
|  | Agree | 26 (19.4%) | 14 (17.7%) |  |
|  | Neutral | 53 (39.6%) | 29 (36.7%) |  |
|  | Disagree | 36 (26.9%) | 25 (31.6%) |  |
|  | Strongly disagree | 2 (1.5%) | 6 (7.6%) |  |
| 1. I believe that the vaccine would allow life to return to normal | Strongly agree | 18 (13.4%) | 9 (11.4%) | 0.854 |
|  | Agree | 56 (41.8%) | 37 (46.8%) |  |
|  | Neutral | 53 (39.6%) | 28 (35.4%) |  |
|  | Disagree | 7 (5.2%) | 5 (6.3%) |  |
|  | Strongly disagree | 0 | 0 |  |
| 1. It is everyone’s social responsibility to get vaccinated, barring any contraindications (eg allergy) | Strongly agree | 35 (26.1%) | 30 (38%) | **0.035** |
|  | Agree | 75 (56%) | 32 (40.5%) |  |
|  | Neutral | 23 (17.2%) | 13 (16.5%) |  |
|  | Disagree | 1 (0.7%) | 4 (5.1%) |  |
|  | Strongly disagree | 0 | 0 |  |
| 1. I prefer the current preventive measures such as social distancing, wearing mask over vaccination. | Strongly agree | 17 (12.7%) | 14 (17.7%) | 0.092 |
|  | Agree | 43 (32.1%) | 33 (41.8%) |  |
|  | Neutral | 54 (40.3%) | 17 (21.5%) |  |
|  | Disagree | 19 (14.2%) | 14 (17.7%) |  |
|  | Strongly disagree | 1 (0.7%) | 1 (1.3%) |  |
| 1. I would be willing to receive a COVID-19 vaccine if it was safe, available and recommended | Yes | 134 (100%) | 76 (96.2%) | **0.023** |
|  | No | 0 | 3 (3.8%) |  |
| 1. I am worried about potential vaccine side effects | Strongly agree | 23 (17.2%) | 10 (12.7%) | 0.337 |
|  | Agree | 60 (44.8%) | 30 (38%) |  |
|  | Neutral | 46 (34.3%) | 33 (41.8%) |  |
|  | Disagree | 5 (3.7%) | 6 (7.6%) |  |
|  | Strongly disagree | 0 | 0 |  |
| 1. I am worried about death or permanent handicap from the vaccine | Strongly agree | 11 (8.2%) | 4 (51%) | 0.313 |
|  | Agree | 21 (15.7%) | 14 (17.7%) |  |
|  | Neutral | 75 (56%) | 37 (46.8%) |  |
|  | Disagree | 26 (15.7%) | 24 (30.4%) |  |
|  | Strongly disagree | 1 (0.7%) | 0 |  |
| 1. I am worried that I would become sick sooner if I were to take the vaccine | Strongly agree | 6 (4.5%) | 1 (1.3%) | 0.315 |
|  | Agree | 24 (17.9%) | 13 (16.5%) |  |
|  | Neutral | 77 (57.5%) | 40 (50.6%) |  |
|  | Disagree | 25 (18.7%) | 23 (29.1%) |  |
|  | Strongly disagree | 2 (1.5%) | 2 (2.5%) |  |
| 1. I am worried about contracting COVID-19 from the vaccine | Strongly agree | 5 (3.7%) | 2 (2.5%) | 0.063 |
|  | Agree | 16 (11.9%) | 7 (8.9%) |  |
|  | Neutral | 69 (51.5%) | 28 (35.4%) |  |
|  | Disagree | 38 (28.4%) | 38 (48.1%) |  |
|  | Strongly disagree | 6 (4.5%) | 4 (5.1%) |  |
| 1. My family would not want me to receive the vaccination | Strongly agree | 4 (3%) | 2 (2.5%) | 0.586 |
|  | Agree | 6 (4.5%) | 6 (7.6%) |  |
|  | Neutral | 75 (56%) | 36 (45.6%) |  |
|  | Disagree | 42 (31.3%) | 31 (39.2%) |  |
|  | Strongly disagree | 7 (5.2%) | 4 (5.1%) |  |
| 1. I am worried that others may refuse to have contact with me If I were to receive the vaccine | Strongly agree | 1 (0.7%) | 2 (2.5%) | 0.377 |
|  | Agree | 2 (1.5%) | 1 (1.3%) |  |
|  | Neutral | 48 (35.8%) | 27 (34.2%) |  |
|  | Disagree | 75 (56%) | 39 (49.4%) |  |
|  | Strongly disagree | 8 (6%) | 10 (12.7%) |  |
| 1. I am worried that others may think that I have COVID-19 if I were to receive the vaccine | Strongly agree | 0 | 0 | 0.320 |
|  | Agree | 8 (6%) | 2 (2.5%) |  |
|  | Neutral | 43 (32.1%) | 20 (25.3%) |  |
|  | Disagree | 70 (52.2%) | 45 (57%) |  |
|  | Strongly disagree | 13 (9.7%) | 12 (15.2%) |  |
| 1. I am concerned about the time that I need to take out of my daily life to receive the vaccine | Strongly agree | 8 (6%) | 6 (7.6%) | 0.187 |
|  | Agree | 50 (37.3%) | 28 (35.4%) |  |
|  | Neutral | 52 (38.8%) | 25 (31.6%) |  |
|  | Disagree | 20 (14.9%) | 20 (25.3%) |  |
|  | Strongly disagree | 4 (3%) | 0 |  |
| 1. I am concerned about having to sign informed consent documents | Strongly agree | 2 (1.5%) | 2 (2.5%) | **0.021** |
|  | Agree | 31 (23.1%) | 20 (25.3%) |  |
|  | Neutral | 75 (56%) | 28 (35.4%) |  |
|  | Disagree | 22 (16.4%) | 27 (34.2%) |  |
|  | Strongly disagree | 4 (3%) | 2 (2.5%) |  |
| 1. It is a hassle for me to request for and receive the vaccine | Strongly agree | 5 (3.7%) | 5 (5.1%) | 0.577 |
|  | Agree | 29 (21.6%) | 22 (27.8%) |  |
|  | Neutral | 70 (52.2%) | 32 (40.5%) |  |
|  | Disagree | 28 (20.9%) | 20 (25.3%) |  |
|  | Strongly disagree | 2 (1.5%) | 1 (1.3%) |  |
| 1. I am concerned over potential allergy to vaccine | Strongly agree | 8 (6%) | 6 (7.6%) | 0.092 |
|  | Agree | 65 (48.5%) | 33 (41.8%) |  |
|  | Neutral | 52 (38.8%) | 27 (34.2%) |  |
|  | Disagree | 6 (4.5%) | 12 (15.2%) |  |
|  | Strongly disagree | 3 (2.2%) | 1 (1.3%) |  |
| 1. Given that immunity may not last long, I am willing to receive repeated COVID-19 vaccinations in my lifetime | Strongly agree | 4 (3%) | 4 (5.1%) | 0.390 |
|  | Agree | 42 (31.3%) | 24 (30.4%) |  |
|  | Neutral | 62 (46.3%) | 28 (35.4%) |  |
|  | Disagree | 23 (17.2%) | 21 (26.6%) |  |
|  | Strongly disagree | 3 (2.2%) | 2 (2.5%) |  |
| 1. Have you suffered from COVID-19? | Yes | 0 | 0 | - |
|  | No | 134 (100%) | 79 (100%) |  |
| 1. If yes, how severe was it? | Required ICU care | - | - | - |
|  | Did not require ICU care | - | - |  |
| 1. Has any of your family members suffered from COVID-19? | Yes | 0 | 0 | - |
|  | No | 134 (100%) | 79 (100%) |  |
| 1. If yes to E3, how severe was it? | Required ICU care | - | - | - |
|  | Did not require ICU care | - | - |  |
| 1. If yes to E3, did any of your family members pass away from COVID-19 infection | Yes | - | - | - |
|  | No | - | - |  |
| 1. Has any of your close friends suffered from COVID-19? | Yes | 0 | 0 | - |
|  | No | 134 (100%) | 79 (100%) |  |
| 1. If yes to E6, how severe was it? | Required ICU care | 0 | 2 (2.5%) | 0.064 |
|  | Did not require ICU care | 0 | 77 (97.5%) |  |
| 1. If yes to E6, did any of your close friends pass away from COVID-19 infection? | Yes | - | - | - |
|  | No | - | - |  |
| 1. Do any of your family members have long-term medical conditions? | Yes | 32 (23.9%) | 29 (37.2%) | **0.039** |
|  | No | 102 (76.1%) | 49 (62.8%) |  |
| 1. Do you have depression or anxiety/would you say that you are an anxious or depressed person? | Yes | 6 (4.5%) | 61 (77.2%) | **≤0.001** |
|  | No | 128 (95.5%) | 18 (22.7%) |  |
| 1. Are you intending to travel (for either business or leisure) after restrictions are lifted? | Yes | 30 (22.4%) | 25 (31.6%) | 0.136 |
|  | No | 104 (77.6%) | 54 (68.4%) |  |

*p*-values≤0.05 are in bold
